# Effects of *Calliandra* and *Sesbania* on Daily Milk Production in Dairy Cows on Commercial Smallholder Farms in Kenya

**DOI:** 10.1155/2020/3262370

**Published:** 2020-02-21

**Authors:** D. N. Makau, J. A. VanLeeuwen, G. K. Gitau, S. L. McKenna, C. Walton, J. Muraya, J. J. Wichtel

**Affiliations:** ^1^Department of Health Management, Atlantic Veterinary College, University of Prince Edward Island, C1A 4P3, Charlottetown, Canada; ^2^Department of Clinical Studies, Faculty of Veterinary Medicine, University of Nairobi, P.O. Box 29053-00625, Nairobi, Kenya; ^3^Department of Applied Human Sciences, University of Prince Edward Island, C1A 4P3, Charlottetown, Canada; ^4^Ontario Veterinary College, University of Guelph, N1G 2W1, Guelph, Canada

## Abstract

There is a growing interest in protein supplementation of dairy-cow diets using leguminous shrubs. The study objective was to ascertain the association between diet supplementation with *Calliandra calothyrsus* and *Sesbania sesban* and milk production in dairy cattle on commercial smallholder farms. This trial involved 235 cows from 80 smallholder dairy farms in Kenya randomly allocated to 4 intervention groups: (1) receiving *Calliandra* and *Sesbania* and nutritional advice; (2) receiving reproductive medicines and advice; (3) receiving both group 1 and 2 interventions; and (4) receiving neither intervention. Farm nutritional practices and management data were collected in a questionnaire, and subsequent physical examinations, mastitis tests, and milk production of cows on the farm were monitored approximately monthly for 16 months. Descriptive and univariable statistical analyses were conducted, and multivariable mixed-model regression was used for identification of factors associated (*P* < 0.05) with daily milk production. The mean milk production was 6.39 liters/cow/day (SD = 3.5). Feeding *Calliandra/Sesbania* to cows was associated (*P* < 0.0005) with an increase in milk produced by at least 1 liter/cow/day with each kg fed. Other variables positively associated with ln daily milk production in the final model included feeding of Napier grass, amount of silage and dairy meal fed, body condition score, and appetite of the cow. Other variables negatively associated with ln daily milk production in the final model included amount of maize germ fed, days in milk, sudden feed changes, pregnancy, and subclinical mastitis. In conclusion, our field trial data suggest that use of *Calliandra*/*Sesbania* through agroforestry can improve milk production in commercial smallholder dairy farms in Kenya. Agroforestry land use systems can be adopted as a way for dairy farmers to cope with feed shortages and low crude protein in farm-available feeds for their cows.

## 1. Introduction

In Kenya, like other East African countries, inadequate nutrition is a major constraint affecting the production and reproduction of dairy cattle [1]. The average daily milk production per cow in most dairy enterprises is estimated to be about 6–7 liters/cow/day which is approximately 70% lower than that of cows in developed countries [2]. This milk production is predominantly attributed to crossbreeds of local breeds (e.g., Brahma, Zebu) with exotic breeds (e.g., Friesian, Jersey, Guernsey, and Ayrshire). Smallholder dairy farms (SDFs) in Kenya constitute an average herd size of 2 cows per farm [2]. While genetics explain some of the difference in the performance of dairy enterprises in developed versus developing countries, management and environmental factors also substantially affect milk production [3].

Traditionally, the impact of inadequate nutrition on dairy farms in Kenya was most felt in the dry season (June–October), where production decreased as good quality feeds dwindled and became more expensive [4, 5]. However, due to changing climatic conditions, wet and dry seasons have become irregular and unpredictable with farmers experiencing longer dry seasons (*personal communication*). Therefore, high costs of feeding dairy cows during these dry seasons progressively lead to high production expenses that hamper growth and profitability of SDFs [6]. The impacts of feed shortage on milk production are exacerbated by inadequate knowledge and technology on feed conservation [7].

To address this production challenge of inadequate nutrition, most farmers prefer to provide home-grown feeds to reduce feed costs; use of crop residues is the most common coping strategy [7]. The crop residue used as the main feed at the peak of the dry seasons for more than 80% of SDFs is dry maize stover. Dry maize stover is a poor-quality feed estimated to have an average crude protein (CP) of 2.5% and neutral detergent fiber (NDF) of 70% [4].

The situation is made worse by an increasing human population and climate change resulting in a decrease in land available for dairy production [8]. To mitigate against this production challenge, integrated sustainable land use models are crucial for smallholder dairy production systems [9]. Agroforestry is a land management system where trees and/or shrubs are combined with crops and/or livestock in the same piece of land [10]. Some of the agroforestry approaches used by farmers include intercropping the fodder trees or shrubs with other crops, or planting them as a hedge during the rainy season and then harvesting in the dry season [11]. Fodder trees can, therefore, play an important role as a feed source to sustain production in livestock and mitigate effects of poor quality feed on milk production, especially in dry season [12].

Some of the fodder shrubs promoted in the highlands of the East African region include *Calliandra*, *Leucaena*, *Chamaecytisus*, and *Sesbania* [13]. Their main advantage (in agroforestry) is the ability to tolerate harsh climatic conditions, such as drought, while providing nutritious animal feed [13]. On average, 2 kilograms of *Calliandra* foliage (dry matter) fed to a dairy cow daily has been reported to increase daily milk production by approximately 1 liter [14]. Prioritization of integrated farming systems with fodder trees and food crops is considered a key step towards sustainable dairy production in Rwanda [15] and perhaps in densely populated rural areas of Kenya as well where there are severe farmland constraints. However, the research on production benefits from leguminous fodder shrubs is primarily found within large-scale or research farms, and therefore these studies do not demonstrate the shrub's benefits on commercial SDFs in Kenya. In this randomized controlled field trial, we monitored a random sample of dairy cows with the aim of ascertaining the association between daily milk production and diet supplementation with *Calliandra calothyrsus* and *Sesbania sesban* in dairy cattle on smallholder farms based on an agroforestry land management model.

## 2. Materials and Methods

### 2.1. Description of Study Area

**Figure 1:**
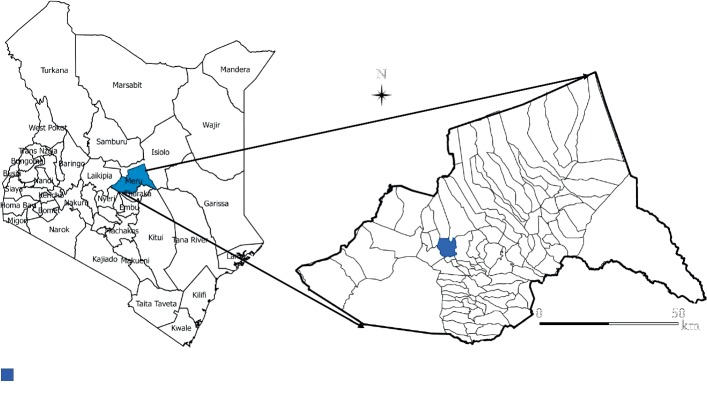
Study area showing Naari sublocation in Meru County, Kenya.

This randomized controlled field trial was carried out in Naari sublocation of Meru County, Kenya (0°6′0″ N and 37°35′0″ E). Meru County is situated on the eastern slopes of Mount Kenya, 270 km north of Nairobi, the capital city of Kenya. Naari sublocation is approximately 2,000 m above sea level. The residents of Naari sublocation mainly practice dairying, subsistence crop farming, horticulture, and lumbering. The most commonly grown crops are food crops, such as maize, beans, and Irish potatoes. The study area was purposively selected because this research was part of a larger study involving dairy farmers in the area (Figure 1) [2, 16, 17]. Moreover, the project partners had a good rapport in the community, providing the necessary goodwill for successful project implementation.

### 2.2. Sample Size and Data Collection

The farmers included in the study were from the Naari Dairy Farmers Cooperatives Society (NDFCS), a dairy group with an active membership of approximately 550 farmers (active member is defined as one who regularly sold milk to the NDFCS at the time of the study). Sample size was based on [18] which conducted a feeding trial on 15 Kenyan cattle and found that milk production was 11.44 and 10.85 kg/day with and without 1 kg (dry matter) of leguminous leaves fed, respectively, with an overall standard deviation of 0.9 kg/day. Using these estimates in a sample size calculation of two independent means, assuming 95% two-sided confidence and 80% power, 38 cows would be needed for each of the intervention and control groups. Assuming that each farm had one cow, the sample size per group was rounded up to 40 to allow for a couple of dropouts. With some farms having more than one cow, it was possible to use a slightly smaller sample size; however, in our experience, a substantial number of cows on SDFs in Kenya have long days in milk with low milk production that is often partially refractory to nutritional attempts to increase milk production. Therefore, it was expected that about 40 cows among the 40 intervention farms would not be refractory to improved nutritional management.

Eighty farms were randomly selected based on inclusion criteria of active membership with the NDFCS, zero-grazing (cows were housed in a free stall area), and <4 milking cows. Out of the 80 farms, 68 of them had more than one cow at a time during the study. All farms were enrolled in the study at the same time. Blinding in this study was not possible since farmers were involved in the management and feeding of the leguminous shrubs, and data collection and analysis was done by the lead author as part of a graduate research project.

The 80 farms were blocked based on days in milk (DIM) and randomly allocated to the intervention and control groups in the field trial. Since changes in milk production due to enhanced feeding are likely to be greater in early lactation, DIM was deemed a very important variable for block randomization. The intervention group was further divided into two with slight modifications where the nutrition intervention only group received leguminous shrubs and nutritional advice, while the nutrition and reproduction (combined) group received leguminous shrubs, reproductive interventions, and nutritional and reproductive advice. Similarly, the control group was divided into two, where the reproduction intervention only group received reproductive interventions and the comparison (control) group did not receive any intervention during the study period. Farmers in the nutrition and combined groups were issued with 150 *Calliandra* and 150 *Sesbania* seedlings to plant on their farms prior to the commencement of the monitoring visits. It was expected that the shrubs would be mature enough to start feeding them to cows at the study commencement. Two types of leguminous shrubs were used since there was a large difference in altitude among the farms in the study area, and it was unclear which type of shrubs would be best on the farms. *Sesbania* is known to be hardier at higher altitudes than *Calliandra* but has slightly lower protein content than *Calliandra* [19, 20]. Farmers receiving nutritional interventions also received advice (in the local language) at each visit on how to feed their cattle better with the feeds and resources available on the farm. Farmers receiving reproduction interventions were provided with advice (in local language) at each visit on better reproductive management and free intrauterine antibiotics (if warranted due to an intrauterine infection recorded) and/or free hormonal injections of prostaglandin F2*α* and/or gonadotropin releasing hormone (if warranted due to intrauterine infection recorded, ovarian cyst recorded, or heat synchronization desired for breeding purposes due to poor heat detection).

Principal farmers in the intervention and control groups consenting to participate in the study were visited approximately monthly and every two months, respectively, from May 2016 to October 2017. During these visits, they responded to a questionnaire adapted and modified from a 2015 baseline study [16]. The questionnaire had sections related to farmer training and demographic information, farm and nutritional management, and cow health and milk productivity.

At these farm visits, physical examinations, including body condition score (BCS—using a 1–5-point scale [21]) and California Mastitis Tests (CMT), were conducted. Cow comfort was also assessed since it can have a substantial impact on milk production [22]. Since cows in the peripartum period and those with DIM >500 days could falsely test positive for mastitis on CMT, positive tests in those cows were only recorded if there was a difference in CMT reaction between quarters. Comfort for each stall was formulated as a composite “comfort score” (with a maximum of 6), which was a function of (1) Stall Length; (2) Stall Width; (3) Stall Lunge Space; (4) Stall Shade; (5) Stall Softness; and (6) Stall Wetness. For comfort score components 1–4, parameters based on cow size were utilized [23]. For comfort score components 5 and 6, knee tests for impact and wetness were utilized, respectively [22, 24]. We included a marginal category to adapt the knee tests to the highly variable stall conditions in Kenya where crop waste and dirt (not sand) are commonly used for floor surfaces.

A score of 1, ½, or 0 was given for each of these 6 individual comfort score components according to the following categorizations: good (equal or surpassed minimum measurements for components 1–4; clearly passing knee tests for components 5–6), fair (within 10% of the minimum measurements for components 1–4; equivocal for components 5–6), or poor (not within 10% of the minimum measurement for components 1–4; clearly failing knee tests for components 5–6). On farms with more than one milking cow, comfort scores for the stalls were averaged, creating one comfort score per farm because cows usually did not lie down in the same stall consistently.

On commencement of the study, farmers were trained on how to weigh quantities of feed fed to the animals once a week and how to record in a provided logbook the feed weights and the daily milk production on the following day after the feed weight measurements were taken. All farmers were issued with standard calibrated spring weighing scales (Spring Balance®) and used large plastic bags for holding quantities of forages for measurement. Although measuring and recording quantities of high protein forages fed was the focus of using weighing scales, weights of other forages were also recorded. The amount of concentrate fed was determined by weighing the filled containers used to measure concentrates when feeding cows on the farms. These entries for each individual cow were averaged at the time of visit to give 1 entry per cow per visit. From anecdotal information obtained during the cross-sectional study in 2015 [16], the feeding regime for each cow was generally quite consistent, at least at the weekly level. Budget and logistical constraints did not allow for laboratory feed analyses; therefore, questions were asked at each visit to categorize the quality of the feed. For example, the height of the Napier grass fed was recorded, since tall Napier grass is known to have low protein content compared to short Napier grass [25].

For farmers who had forgotten to record the milk and feeding details in the logbook during the period since the last visit, milk production for the visit was assessed based on the previous day's total milk production for the cow. Feed weights were also assessed based on the current portions being fed to the cow on the day prior to the visit. At each cow visit, all farmers were asked if the production and feeding on the day prior to the visit were representative of the production and feeding for each cow since the last visit, and in most cases, 83.3% of the cow visits (1214/1458), the farmers confirmed that production and feeding were representative. Therefore, data collected on the date of the visit were assumed to be representative of the monthly management and production. For the 16.7% of cow visits when there were discrepancies, average measurements between available logbook recordings and current measurements were used to minimize reporting bias which was likely in this type of study [22].

### 2.3. Data Management and Analysis

Field data were entered into MS Excel 2010 (Microsoft, Sacramento, California, USA). Statistical analyses were done using Stata13.0 software (StataCorp LLC, College Station, Texas, USA). There was a hierarchical nature to the data, with visits clustered within cows and cows clustered within farms.

Descriptive statistics included means, medians, distributions, and proportions, where applicable. The Shapiro–Wilk test for normality of daily milk production outcome was significant (*P* < 0.05), and a histogram of the outcome had a positive skew of 1.3, and therefore suitable transformations for the outcome were explored. This skewed distribution of the outcome was addressed by transforming it onto the natural log scale for purposes of parametric statistical analyses. For ease of interpretation of statistical analyses utilizing this transformed outcome, the outcome was transformed back to the normal scale.

The primary study objective was to determine if the nutrition intervention contributed to higher daily milk production on those farms receiving the intervention. Since the combined group also received reproductive intervention which could also improve daily milk production if cows calved out more quickly than cows on farms in non-reproduction intervention groups, combining the two nutrition intervention groups and two non-nutrition intervention groups together in the statistical analyses was not desired. As a result, significant differences in natural log of daily milk production between the four intervention groups were assessed using the Bonferroni-adjusted one-way ANOVA.

These analyses would assume that the random allocation to groups balanced out the other factors (confounders) that may affect daily milk production between groups (Table 1). At the start of the study, other known factors that may affect daily milk production (e.g., weight, height, age, body condition score, pregnancy status, and parity) were compared by group using ANOVA or Fisher's exact analyses to confirm that groups were not different for these variables. Nonsignificant *P* values were confirmatory that the random allocation was successful at balancing these factors among groups.

As a secondary study objective, subsequent data analyses (modeling) were also conducted based on the actual feeding of the cows by the farmers as opposed to the different study group classifications because of lack of intervention compliance. Noncompliance of a farmer was mainly related to feeding and data-recording recommendations. During each of the visits, 5–10% of recommendations would not be implemented completely. Noncompliance was when a farmer had neither weighed the amount of fodder and other feed given to the cow nor recorded the milk production of the cow. Partial compliance included keeping the records for only part of the period between farm visits, weighing only some of the fodder and no other feeds, and/or not feeding the recommended weight of shrub foliage. Reasons for partial or noncompliance among farmers included (1) inadequate foliage (especially in the dry season); (2) poor harvesting technique; (3) transition between hired helps; (4) farmer illness; and (5) other personal reasons. Growth and regeneration of the leguminous shrubs was largely dependent on the farmers' management practices (weeding, watering, manure use, etc.) and prevailing weather conditions. Since farmers were consistently encouraged to comply with the project guidelines, farmers were rarely noncompliant for two consecutive visits.

For these subsequent statistical analyses, some cows had missing DIM (6% of 1458 cow visits) because the farmers had bought cows into the farm and they had not obtained the reproductive history of the cows from the seller. Therefore, DIM data was presumed to be missing completely at random (MCAR) and imputation would be beneficial for modeling purposes. For these purchased cows with missing DIM data, the overall mean DIM was inserted as an imputation to avoid loss of observations in the models [26].

A univariable mixed linear regression model with restricted maximum likelihood (REML) was fitted for each of the predictor variables to ascertain associations with natural logarithm of daily milk production (*P* ≤ 0.4). Factors significant at *P* ≤ 0.4 and other suspected confounders were considered for a mixed multivariable model-building process. Tests for correlation (Pearson's correlation coefficient) among all parameters meeting the regression modeling cut-off (*P* ≤ 0.4) were done to aid decision-making for model-building when it came to which highly correlated variables should be included in the model. For highly correlated predictors, the predictor that was included in the models was decided based on biological plausibility.

Multivariable mixed linear regression (REML) was subsequently performed with natural logarithm of daily milk production in liters as the outcome (*P* ≤ 0.05). Two models were fitted to account for the two different levels of clustering, both through the inclusion of random effects. Model 1 controlled for clustering at the visit level, while model 2 controlled for clustering at the cow level.

Model 1 was fitted with an autoregressive correlation (ar) structure assuming that the correlation between 2 contiguous visits would be exponentially greater than 2 noncontiguous visits [27, 28].

Model 1 *(random effect of visits clustered within cows)*: *Ln of milk/cow/day* *=* *constant* *+* *amount of dairy meal fed (kg)* *+* *amount of maize silage (kg)* *+* *amount of Calliandra/Sesbania fed (kg)* *−* *amount of Calliandra/Sesbania fed squared (kg)* *−* *amount of maize germ fed (kg)* *+* *BCS–BCS squared* *−* *DIM* *+* *DIM squared* *+* *normal appetite* *−* *pregnant status–subclinical mastitis* *−* *sudden feed changes*.

Model 2 was fitted with an exchangeable correlation structure. The assumption was that the correlation between any two cows within a farm was the same [27, 28]. Model AIC was used to select the best final model with the appropriate correlation structure.

Model 2 *(random effect of cows clustered within farms)*: *Ln of milk* *=* *constant* *+* *visit number* *+* *amount of dairy meal fed* *+* *amount of Calliandra/Sesbania fed* *−* *amount of maize germ fed* *+* *amount of maize silage* *+* *feeding Napier grass* *+* *BCS* *−* *BCS squared* *−* *DIM* *+* *DIM squared* *+* *normal appetite* *−* *pregnant status* *−* *sudden feed changes*.

Interactions between significant model fixed effects were explored. Wald's test was used to test overall significance of categorical parameters with more than 2 categories. Assessment of linearity between daily milk production and continuous variables was done using LOWESS plots for visualization. Variables with nonlinear relationships with the outcome were fitted as curvilinear terms in the model, where applicable.

Model-building was done through the manual backward stepwise elimination technique, and *P* values were used to determine fixed effects to keep in the model. None of the model parameters had a correlation higher than 0.4. Testing for suspected confounding of model variables by variables not in the final model was done by comparing changes in coefficient estimates with and without the suspected confounders. Model evaluation was done to confirm that normality and homoscedasticity assumptions on both random and fixed effects were met. Identification of extreme and influential observations was done by graphing the standardized residuals and comparing changes in coefficient estimates and their significance when modeling with and without influential observations. Predictions of daily milk production were performed on a back-transformed scale of milk produced in liters/day.

## 3. Results

In this trial, a total of 607 visits were made to 80 farms on which 235 cows were included in the study. Only a portion of these cows was milking at any given visit, generating 1458 cow-visit observations during the study period (16 months). Observations when cows were not milking/dry were excluded from the analysis.

**Figure 2:**
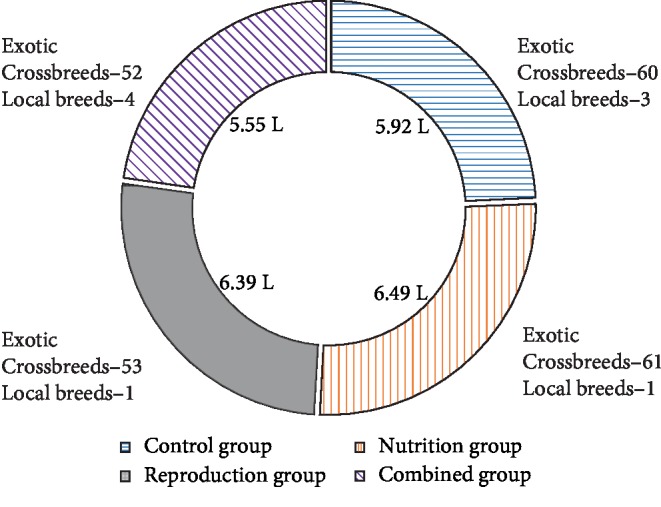
Mean daily milk production in liters (L) per cow and farm demographics.

The mean milk production/cow/day was 6.39 liters (s.d. 3.5) with a median of 6.0 liters and a range of 0.25–27.5 liters. Cows kept on the trial farms were predominantly exotic (i.e., Friesian, Ayrshire, Jersey, and Guernsey) crosses (Figure 2) and therefore breed did not affect daily milk production (*P* > 0.05).

For the primary study objective of comparing group outcomes, there were no significant (*P* > 0.05) differences in natural logarithm of daily milk production between the four intervention groups using the Bonferroni-adjusted one-way ANOVA. Therefore, the results from the second set of statistical analyses were essential for determining the impact of the use of the *Calliandra* and *Sesbania* shrubs in the nutritional management of the cows, considering compliance levels of what was actually fed.

### 3.1. Descriptive Statistics and Univariable Analyses between Natural Log of Milk Production and Various Factors

Several variables met the *P* ≤ 0.4 cut-off on univariable mixed linear regression analyses for associations with natural logarithm of daily milk production when accounting for clustering of visits within cows. Differences in the natural log of daily milk production for these variables among the 4 study groups are shown in Tables 2–4, although these differences could be confounded by other variables and therefore should be interpreted in conjunction with the multivariable model results. In order to provide further description of the trial population, overall means/proportions and significant group differences in percentages or means for these variables are described here.

Farmers mostly used dairy meal as the concentrate feed, and only seldom used maize germ (Table 2). Farmers generally maintained consistent concentrate feeding regimes to cows on their farms despite changes in DIM or milk production. Napier grass was fed for most of the study period, and farmers preferred to feed any available Napier grass at any height rather than not feed any Napier grass at all (Table 2). Cows in 14–20% of the farms experienced sudden changes in feed, which was comparable among the groups. Most of the study period (>64% of visits) was characterized by dry weather (Table 2). The mean amount of dairy meal fed was 1.6 kg/cow/day, while maize germ was fed at much smaller quantities (0.03 kg/cow/day, Table 4). Farmers in the 2 nutrition intervention groups were providing significantly more mineral/vitamin supplements than the reproduction group (Table 4). Maize silage was used by some farmers (13.4%) to support milk production, with average amounts of 2.0 kg/cow/day being fed across all farms and 3.3 kg/cow/day being the highest amount fed to the animals.

Of the two nutrition intervention groups, the nutrition only group fed 0.1 kg more *Calliandra* and *Sesbania* than the combined intervention group (Table 4), with both groups feeding significantly more (*P* < 0.0005) than the other two groups. Farmers also used other supplementary feed (bean pods, vegetables, kitchen byproducts, wheat straw, etc.) as a coping strategy to supplement diets of cows when there were feed shortages. The level of this stopgap feeding practice was significantly higher (*P*=0.0006) in the comparison group (0.6 kg/cow/day) than the other 3 groups (Table 4).

For most of the visits, cows in all groups were healthy with physical exam parameters within normal ranges; cows had normal appetite more than 98% of the time across all groups. There were significant differences (*P*=0.021) in proportions of examined animals affected by skin parasites (ticks) during the visits, with more infestation observed in the reproduction group than the other groups (Table 3) for most of the study period. Subclinical mastitis occurrence was highest in the nutrition group (21.9%) and lowest in the reproduction group (11.2%) (Table 3) (*P*=0.043). Pregnancy risks were relatively low and similar across all groups (*P* > 0.05).

The mean BCS of cows was quite similar among groups (Table 4), with the overall mean being 2.2 out of 5. The mean DIM for cows in the nutrition and combined groups was 299.3 (s.d. 10.8) days and 249.4 (s.d.10.5) days, respectively, but these DIM were not significantly different from the other groups. Pen comfort scores were similar across groups (Table 4), with the overall average being 3.1 out of 6.

### 3.2. Multivariable Analysis between Natural Log Daily Milk Production (Liters) and Various Factors

The multivariable mixed linear regression models with the natural log of daily milk production as the outcome variable were based on 1458 cow-visit observations from 607 farm visits of 235 cows on 80 farms. There was an average of 6.2 visits/cow, with a maximum of 16 visits/cow. There was an average of 1.6 cows/farm/visit with a maximum 3 cows/farm/visit, as dictated by the inclusion criteria. In the evaluation of correlation among eligible model variables, there were various predictors with correlation of more than 0.4. However, none of the correlated variables remained significant in the final multivariable model, and therefore decision-making among correlated variables for the final model was unnecessary.

**Figure 3:**
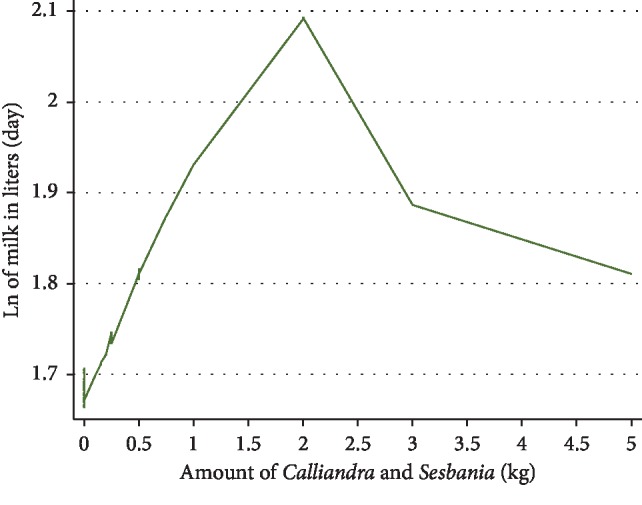
LOWESS plot indicating a curvilinear relationship between amounts of *Calliandra/Sesbania* fed and natural log of milk production/day for 1458 cow-visit observations from 607 farm visits of 235 cows on 80 smallholder dairy farms near Meru, Kenya, in 2016–2017.

**Figure 4:**
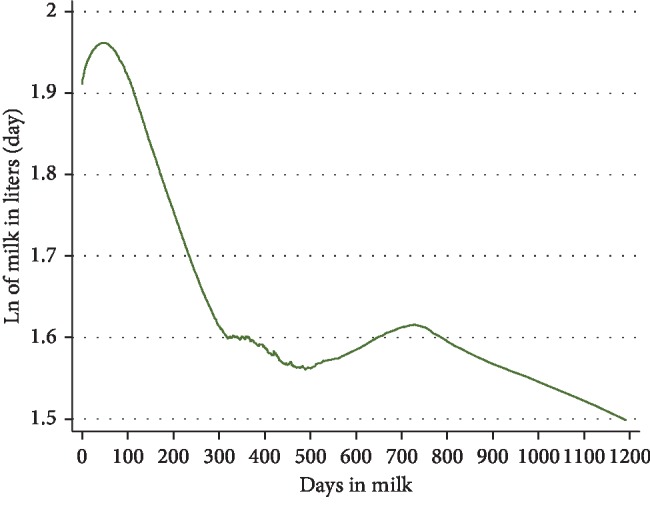
LOWESS plot indicating a curvilinear relationship between days in milk and natural log of milk production/day for 1458 cow-visit observations from 607 farm visits of 235 cows on 80 smallholder dairy farms near Meru, Kenya, in 2016–2017.

**Figure 5:**
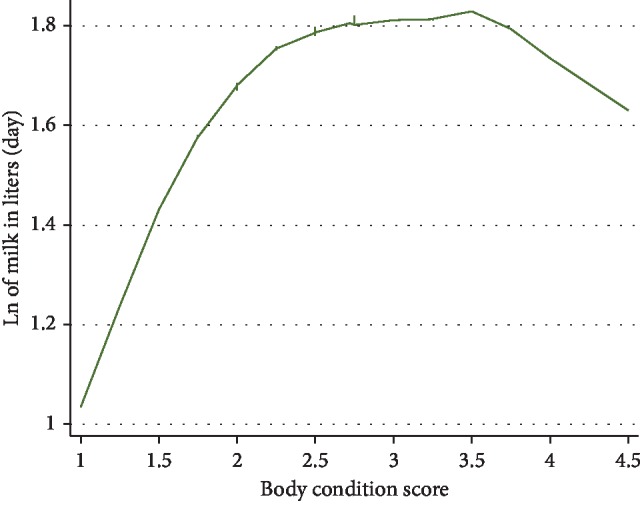
LOWESS plot indicating a curvilinear relationship between body condition and natural log of milk production/day for 1458 cow-visit observations from 607 farm visits of 235 cows on 80 smallholder dairy farms near Meru, Kenya, in 2016–2017.

In the first final multivariable linear mixed model controlling for clustering of visits within cows, normal appetite of the animal, BCS, and amounts of dairy meal, *Calliandra/Sesbania*, and maize silage fed to cows were significantly positively associated with amount of milk/cow/day produced on the natural log scale. Factors negatively associated with natural log of daily milk production were the amount of maize germ fed, DIM, pregnancy status of the animal, whether the animals had subclinical mastitis or sudden changes in their feeding. Amount of *Calliandra/Sesbania* fed (Figure 3), BCS (Figure 4), and DIM (Figure 5) were all curvilinearly associated with natural log of daily milk production. Feeding 2 kg (wet weight) of *Calliandra/Sesbania* appeared to have the optimum effect of increasing milk production, associated with 50% higher milk production when compared to feeding no *Calliandra/Sesbania* (Figure 3). However, on occasion, a few farmers with lower milk production were able to feed ≥2.5 kg of foliage to their cows per day, affecting the shape of the graph. Milk production was estimated to peak within the first 100 DIM, as expected, before consistently decreasing for the rest of the lactation period, with a small number of cows in late lactation (500–900 DIM) with slightly increased milk production on farms with more abundant higher-quality feed being fed to these late-lactation cows (Figure 4). The highest milk production was observed when BCS was 3.5 (Figure 5). However, on rare occasions, cows had BCS more than 3.5, but they were not accompanied by better milk production (Figure 5) because their DIM was high (>275 days).

Interpreting the exponentiated coefficients of factors not in figures, when all factors were held constant and accounting for clustering of visits within cows, a kg increase in the amount of dairy meal (between 0 and 7 kg) fed was estimated to result in a 3.9% increase (*P* < 0.0005) in mean amount of milk produced/day (Table 5). Mean milk production for cows increased by 0.8%/cow/day with every kg increase in maize silage fed (between 0 and 30 kg). However, feeding maize germ to cows significantly resulted in reduced mean milk production/day (i.e., with every kg increase of maize germ, there was a 27.1% decrease in milk production/cow/day) (Table 5). When abrupt changes were made to the cow's diet, the mean milk production/day was estimated to decrease by 9.9%. When cows had a normal appetite, mean daily milk production was two times higher (*P* < 0.0005) compared to when appetite was poor. When a cow was pregnant or with subclinical mastitis, mean milk production/day for the cow at that time was reduced by 23.4% and 6.0%, respectively (Table 5). The estimated within-group correlation for observations in this model was 0.376.

In the second final multivariable linear mixed model controlling for clustering of cows within farms, the natural log of milk production of a cow/day was observed to be higher by 0.9% in every subsequent visit after the first visit (Table 6). There were many similarities in this final model compared to the model controlling for clustering of visits within cows, with only minor differences in coefficients. There were three substantive differences between models. Feeding Napier grass was not significant in the first final model but had a significant positive association with ln milk production/cow/day. Conversely, subclinical mastitis and the quadratic form of *Calliandra/Sesbania* were not significant and so were not included in this second final model. In this model, the estimated within-group correlation for the observations was 0.464.

### 3.3. Model Evaluation

For both models, the model assumptions on normality and homoscedasticity were met on the farm, cow, and visit levels. Scatter plots of fitted values and standardized residuals also did not depict distinct patterns in the distribution of standardized residuals at all levels of the model. Any standardized residuals greater than 2 standard deviations were found not to be true outliers. The standardized residuals had a good fit on the normality plot. Removal of these observations had no effect on significance and coefficients of the predictors with one exception; there was an 8% decrease in the effect of subclinical mastitis on natural log of milk without affecting its significance in model 1 and a 12% decrease in the effect of sudden feed changes on natural log of milk without affecting the variable significance in model 2. These observations were influential but not true outliers; therefore, all observations were retained in both final models.

## 4. Discussion

Our field trial data suggest that feeding *Calliandra*/*Sesbania* along with nutritional advice can be used to improve daily milk production on commercial SDFs (Tables 5 and 6). As such, agroforestry land use systems can be adopted as a way for dairy farmers to cope with feed shortages and low crude protein in farm-available feeds for their cows. Daily milk production on the study SDFs improved even when no direct nutritional interventions were used on the farms (i.e., towards the end of the study, two farmers in the reproduction group grew the shrubs on their own, harvested them when ready, and fed them to their cows). Farmers sometimes communicate on farm management practices with each other in these close-knit communities in Kenya.

These leguminous shrubs utilized in the trial are high in protein and, therefore, supplement the CP necessary for good milk production in dairy cows feeding on poor-quality feed [13, 18, 23]. This trial result was similar to findings observed on SDFs in another part of central Kenya [29] where milk production was observed to increase by 0.4 kg/day when *Calliandra/Sesbania* was fed to a cow (amounts were not measured in that study).

The negative effect of feeding maize germ on daily milk production was unexpected (Tables 5 and 6). Very few farmers were using maize germ as a concentrate supplement (*n* = 11), leading to very small amounts being fed. Some farmers who used maize germ chose to formulate their homemade concentrate mix by combining some maize germ with dairy meal, bran, and/or other available grains, producing an economical but lower protein mix than dairy meal alone. Consequently, farmers feeding maize germ (especially in comparison group) effectively reduced the amounts of protein supplements in the cow's diet, resulting in a negative effect of maize germ in the final model. This situation arises when nutrients are not balanced due to inadequate knowledge of the farmer to properly formulate a ration [30].

While the shrubs provide an excellent source of crude protein, we know that an adequate balance of energy and proteins are necessary for milk production [31]. Irrespective of which level of clustering was controlled for in the present study, daily milk production increased when more dairy meal was used to supplement the animals' diets. The dairy concentrate findings of this study were in agreement (although lower) with several other studies [5, 29, 32, 33]. The small increase in milk production was likely a function of the poor BCS of study cows and small amount of dairy concentrate being fed in the study area (Table 4).

In this trial, an increase in mean milk production in liters/cow/day was observed with an increase in the amount of silage fed (Tables 5 and 6). Maize silage in this area was mostly made of whole maize plants harvested at the “milk” stage. Other additives included during silage preparation were wheat bran, molasses, and/or urea, depending on the preference, accessibility, and availability of these products to farmers. These additives are generally aimed at improving the available protein [34] and metabolizable energy (ME) content [35] and supporting the fermentation process [36] in silage. Similar practices have been documented elsewhere [37]. It was therefore expected that when farmers added maize silage to the daily cow ration, this would provide additional CP and energy necessary for better milk production. Well-prepared whole maize silage also has a low NDF proportion, leading to increased digestibility and higher dry matter intake (DMI), which would support an increase in milk production [38].

Feeding Napier grass to cows in this study population was associated with higher daily milk production (Table 6). Irrespective of what height of the Napier grass was fed, feeding Napier grass was significantly better (7.6% increase in mean milk production/day) than not feeding any Napier grass. Napier grass provided additional CP and energy [39] to the cows over and above what was received through other diets fed to the cows, thus improving milk production, as observed elsewhere [40]. The effect of feeding Napier grass was, however, not significant in model 1 (when accounting for clustering of visits within cows), perhaps because other factors influenced the direct effect of Napier grass fed on milk production at given times of the study. There was no evidence of confounding in the final model, so these other factors could have been practices or factors that were not consistent throughout the study period. Therefore, by having visits within cows as a random effect, some monthly variability in daily milk production attributed to Napier grass was accounted for in the random part of the model, making Napier grass insignificant in the fixed part of the model.

A higher BCS was significantly associated with better daily milk production in these cows (Tables 5 and 6), which was in agreement with other studies [29, 41]. Poor body condition is indicative of a current or previous negative energy balance in a cow [41], which affects milk production, milk composition, and reproduction of dairy cows [42]. This imbalance is a common occurrence on SDFs in developing countries during the dry season when the only readily available feed is often of poor quality [1, 4]. Feeding high-quality preserved forages such as hay or silage is recommended for these dry season periods.

There were only a few animals producing milk with DIM greater than 500, although the mean DIM of 285 was higher than desired. The unexpected shape of the graph (Figure 4) depicting an increase in daily milk production for very high DIM is specific to those few cows and their cow- and farm-level characteristics. This production curve was similar to other studies that depicted the physiological norm of daily milk production in dairy cows with peak production experienced about 2 months postpartum [43–45].

Higher daily milk production was observed when animals had a good appetite (Tables 5 and 6). This finding is similar to other studies where higher DMI was associated with better milk production since such animals would have the necessary CP and ME for higher milk production [46, 47]. Better appetite would certainly complement the effect of increased milk production from feeding the shrubs, while poor appetite would negate this positive effect.

Being pregnant was associated with a reduction in mean daily milk production in this study population (Tables 5 and 6). Pregnant cows spend more of their energy to support pregnancy and consequently reduce the amount of milk produced [48–50]. In some cases, farmers said they even chose to reduce frequency of milking when pregnant cows seemed to lose body condition too much, which would also lead to lower daily milk production. This decision was subjective and was made independently by the farmer which may have led to a reduced difference in milk production between farms in the combined intervention group and other groups.

Subclinical mastitis was significant in model 1 when accounting for clustering of visits within cows but was not significant in model 2 when accounting for clustering of cows within farms. Controlling for visits within cows allowed for the effects of new cases in cows to be accounted for in the fixed part of the model. Subclinical mastitis was associated with a 6% decrease in daily milk production, on average, compared to when there was no subclinical mastitis, which is similar to another study in SDFs in Kenya [29]. Inflammation of mammary tissue and damage by mastitis-causing pathogens resulted in reduced milk secretion and letdown [50]. This effect may have negatively affected the observed impact of increased milk production from the cows' diet supplementation with the shrubs.

When farmers abruptly changed the diets of dairy cows, daily milk production decreased significantly (Tables 5 and 6). This reduction was likely as a result of reduced feed intake and metabolic upset as the cows' rumens adapted to the new diet introduced. This effect would decrease the positive effect of the shrubs on milk production.

Although our study design was aimed at controlling for confounding by various factors through multivariable regression analyses, the study period was long (16 months) and so the study population changed with time. New cows were introduced into the farms (purchased) and added to the study, while other cows left the study (open population), and this could have contributed to some selection bias for the make-up of the four groups.

There was some loss to follow-up in the study, which may also have affected our study results. However, the withdrawals from the study were determined to be not for study-related reasons. The reasons included death of the cow or changes in farmer priorities, among other family issues. Therefore, it is unlikely that these withdrawals led to biased results.

The allocation of 20 farms to four groups was based on the intended intervention of *Calliandra/Sesbania* foliage being fed to cows in the 2 intervention groups (nutrition and combined), with the hypothesis that the combined group would experience a synergistic positive effect on conception (results in a related publication [51]). Performance of these shrubs was variable and largely dependent on management, prevailing weather conditions, and availability of water for the shrubs. Due to the practical challenges associated with growing the shrubs, farmers in the intervention groups did not all feed equal amounts of the shrub foliage all the time, either due to lack of foliage, poor harvesting technique, or lack of compliance. For this reason, the second set of data analyses was based on the actual feeding practices of the farmers as opposed to the different study groups. These practical challenges of interventions on privately owned farms in the target population are part of the rationale to determine intervention effects in field trials rather than research farms.

Well-designed randomized controlled trials are powerful tools for evaluating the impact of different farm interventions [52]. However, they are complex and when done in field settings, these studies can be plagued by many challenges and our study was no exception. Variation across farming practices complicated our data collection and statistical analyses, but this variation was an asset of the study because we wanted to determine the benefits of feeding leguminous shrubs on the breadth of smallholder farms in this area of Kenya, which will be more representative of what farmers should expect compared to the studies on large or research farms. The variability in on-farm data recording was also a limitation of our study, and the variability in literacy levels of the farmers contributed to some of the recording variability—realities of doing research among smallholder dairy farmers in Kenya. Both study design and choice of analytical tools were aimed at mitigating the implications of the aforementioned limitations on the interpretation of the study results. While acknowledging the shortcomings of this study, these results indicate that the effects of *Calliandra* and *Sesbania* supplementation observed in studies done on large-scale or research farms can be effectively translated to SDFs as observed in our intervention approach.

Future research would benefit from a nutrient analysis of the different homemade concentrate mixes that farmers used on their farms (e.g., when maize germ was fed) to better quantify the effect of this farm practice. Research should also be done on the financial implications of using an agroforestry model to feed *Calliandra* and *Sesbania* to cows, which would elucidate the sustainability and possible wider adoptability of this intervention by SDFs in Kenya. Ecologically, an assessment of the effects of long-term use of *Calliandra* and *Sesbania* on soil structure and properties would also be beneficial to farmers, since some earlier literature [53] positively associated *Sesbania* shrubs with some species of soil nematodes, which may affect other food crops on the farms in the long term.

## 5. Conclusions

From the findings of our study, we conclude that with proper nutritional advice, diet supplementation with C*alliandra* and S*esbania* can improve daily milk production in commercial SDFs in Kenya. Agroforestry land use systems can be adopted as a way for dairy farmers to cope with shortages and poor quality in farm-available feeds for their cows. Concentrate feeding (dairy meal) to dairy cows was shown to improve daily milk production and should be fed optimally. However, improper homemade mixes of dairy concentrates, as shown with maize germ feeding, may result in lower daily milk production. Therefore, advice on homemade mixes should be sought from skilled personnel. Although shorter Napier grass is more nutritious than tall mature Napier grass, feeding Napier grass at any height is better than not feeding any at all, and so farmers should continue feeding Napier grass, when available, rather than poor-quality feeds such as dry maize stover. Smooth transition when changing feed for dairy cows would be a better way to ensure consistent daily milk production rather than abrupt changes; hence, inventory management and better feed delivery planning is required on these farms. Better BCS and shorter DIM would be more profitable to the farmers since cows would produce more milk; therefore, attention is needed to improve reproduction and adopt the feeding recommendations mentioned above. Subclinical mastitis affects daily milk production and thus farmers should utilize CMT for early detection and treatment of mastitis cases while employing preventive management practices that reduce occurrences of new cases of mastitis on their farms. Overall, this project had a positive impact on SDFs in this study area, given the improved daily milk production observed over the different farm visits compared to the baseline.

## Figures and Tables

**Table 1:** Distribution of animal parameters among different groups prior to the intervention for 80 smallholder dairy farms near Meru, Kenya, in 2016.

Parameter	Comparison group mean (s.d.)	Nutrition group mean (s.d.)	Combined group mean (s.d.)	Reproduction group mean (s.d.)	ANOVA *P* value
Height (cm)	124.3 (19.7)	115.9 (7.0)	119.0 (7.3)	118.6 (6.7)	0.165
Weight (kg)	387.8 (77.3)	383.7 (71.6)	391.9 (60.1)	395.6 (59.5)	0.922
Body condition score	2.2 (0.5)	2.1 (0.5)	2.2 (0.5)	2.2 (0.6)	0.625
Age (years)	5.8 (2.0)	5.5 (2.0)	5.5 (2.2)	5.8 (2.5)	0.949
Parity	2.5 (1.3)	2.5 (1.6)	2.7 (1.5)	2.7 (1.5)	0.932
Pregnant (%)	38.7% (12/31)	40.6% (13/32)	25.0% (8/32)	38.9% (14/36)	0.848^*α*^

^*α*^ *P* value from Fisher's exact test.

**Table 2:** Descriptive statistics for farm-visit level categorical variables for unconditional mixed linear regressions for variables with *P* ≤ 0.40 associations with natural log of daily milk production from 607 farm-visit observations to 80 smallholder dairy farms near Meru, Kenya, in 2016–2017.

Variable and categories	Percentage in comparison group (*n* = 95 farm visits)	Mean daily milk production (liters)	Percentage in nutrition group (*n* = 219 farm visits)	Mean daily milk production (liters)	Percentage in combined group (*n* = 199 farm visits)	Mean daily milk production (liters)	Percentage in reproduction group (*n* = 94 farm visits)	Mean daily milk production (liters)	*P* value for difference in ln milk production
Concentrate supplementation									0.184
Yes	70.5% (67)	6.0	76.7% (168)	6.7	77.4% (154)	6.5	71.3% (67)	5.8	
No	29.5% (28)	5.8	23.3% (51)	5.9	22.6% (45)	5.9	28.7% (27)	4.9	
Changes in concentrate amounts									0.091
Yes	6.0% (4)	8.6	18.5% (31)	7.0	16.2% (25)	6.1	11.9% (8)	6.2	
No	94.0% (63)	5.7	81.5% (137)	6.4	83.8% (129)	6.4	88.1% (59)	5.4	
Napier grass fed									0.002
Yes	85.3% (81)	6.2	82.2% (180)	6.4	82.4% (164)	6.5	70.2% (66)	5.8	
No	14.7% (14)	4.5	17.8% (39)	6.8	17.6% (35)	5.9	29.8% (28)	5.1	
Sudden change in feed									0.002
Yes	13.8% (13)	5.9	18.7% (41)	6.4	19.1% (38)	6.4	13.8% (13)	4.7	
No	86.2% (82)	5.9	81.3% (178)	6.5	80.9% (161)	6.4	86.2% (81)	5.7	
Season									0.033
Dry	78.9% (75)	5.8	74.9% (164)	6.6	75.4% (150)	6.4	62.8% (59)	5.7	
Wet	21.1% (20)	6.6	25.1% (55)	6.1	24.6% (49)	6.5	37.2% (35)	5.4	

Reproduction farms were visited as frequently as the nutrition and combined farms. However, some farm visit entries on reproduction farms were removed from the model analysis since these farms only had dry cows on these occasions (14%).

**Table 3:** Descriptive statistics for cow-visit observation level categorical variables for unconditional mixed linear regressions for variables with associations with natural log of daily milk *P* ≤ 0.40production for 1458 cow-visit observations in 235 cows on 80 smallholder dairy farms near Meru, Kenya, in 2016–2017.

Variable and categories	Percentage in comparison group (*n* = 227 cow-visit observations)	Mean daily milk production (liters)	Percentage in nutrition group (*n* = 434 cow-visit observations)	Mean daily milk production (liters)	Percentage in combined group (*n* = 432 cow-visit observations)	Mean daily milk production (liters)	Percentage in reproduction group (*n* = 365 cow-visit observations)	Mean daily milk production (liters)	*P* value for difference in ln milk production
Normal appetite									0.001
Yes	98.7% (224)	6.0	99.5% (432)	6.5	99.0% (428)	6.4	99.5% (363)	5.6	
No	1.3% (3)	5.5	0.5% (2)	0.0	1.0% (4)	1.0	0.5% (2)	2.0	
Skin parasites present									0.128
Yes	90.7% (206)	5.4	84.8% (368)	6.4	84.0% (363)	6.4	96.7% (353)	5.6	
No	9.3% (21)	9.3	15.2% (66)	7.1	16.0% (69)	5.6	3.3 % (12)	5.5	
Subclinical mastitis									0.131
Yes	20.3% (46)	6.1	21.9% (95)	5.6	14.8% (64)	8.0	11.2% (41)	4.1	
No	79.7% (181)	5.9	78.1 % (339)	6.8	85.2% (368)	6.1	88.8% (324)	5.7	
Pregnant									<0.0005
Yes	26.0% (59)	5.2	27.6% (120)	5.2	24.1% (104)	5.6	26.3% (96)	4.9	
No	74.0% (168)	6.2	72.4% (314)	7.0	75.9% (328)	6.6	73.7% (269)	5.8	

**Table 4:** Descriptive statistics for continuous variables from unconditional mixed linear regressions with *P* ≤ 0.40 associations with natural log of daily milk production for 1458 cow-visit observations from 607 farm-visits for 235 cows on 80 smallholder dairy farms near Meru, Kenya in 2016–2017.

Variable names	Mean (s.d.) in comparison group (*n* = 227 cow observations)	Mean (s.d.) in nutrition group (*n* = 434 cow observations)	Mean (s.d.) in combined group (*n* = 432 cow observations)	Mean (s.d.) in reproduction group (*n* = 365 cow observations)	*P* value for difference in ln milk production
Amount of daily dairy meal (kg)	1.41 (0.21)	1.64 (0.12)	1.75 (0.11)	1.62 (0.09)	<0.0005
Amount of daily maize germ (kg)	0.04 (0.01)	0.00 (0.00)	0.03 (0.01)	0.01 (0.01)	0.010
Amount of daily mineral/vitamin (g)	38.59 (1.43)	39.83 (0.95)	40.60 (0.46)	30.00 (1.48)	<0.0005
Amount of daily *Calliandra/Sesbania* (kg)	0.00 (0.00)	0.15 (0.02)	0.11 (0.02)	0.01 (0.01)	0.002
Amount of other supplementary feed (kg)	0.56 (0.54)	0.36 (0.35)	0.27 (0.27)	0.07 (0.03)	0.071
Amount of daily maize silage (kg)	1.90 (0.56)	1.57 (0.30)	1.35 (0.34)	3.32 (0.68)	<0.0005
Body condition score	2.17 (0.08)	2.19 (0.04)	2.24 (0.04)	2.29 (0.04)	<0.007
Days in milk	313.0 (14.6)	299.3 (9.7)	248.3 (8.7)	291.6 (12.0)	<0.0005
Stall comfort score^*∗*^	2.68 (0.51)	3.02 (0.35)	2.91 (0.31)	3.48 (0.10)	0.102

^*∗*^Farm-visit level variable based on farm-visit numbers by group: comparison group *n* = 95 farm visits, nutrition group *n* = 219 farm visits, combined group *n* = 199 farm visits and reproduction group *n* = 94 farm visits.

**Table 5:** Final generalized linear mixed regression model for natural log of daily milk production for 1458 cow-visit observations from 607 farm-visits of 235 cows on 80 smallholder dairy farms near Meru, Kenya in 2016–2017, adjusting for clustering of visits within cows.

Variables and their categories	Exponentiated coefficient	Coefficient	(95% conf. Interval)		*P* value
Amount of daily *Calliandra/Sesbania* (kg)	1.376^*β*^	0.319^*β*^	0.174^*β*^	0.464^*β*^	<0.0005^*β*^
Amount of daily *Calliandra/Sesbania* squared (kg squared)	0.927^*β*^	−0.076^*β*^	−0.127^*β*^	−0.025^*β*^	0.003^*β*^
Amount of daily dairy meal (kg)	1.039	0.038	0.018	0.057	<0.0005
Amount of daily maize germ (kg)	0.729	−0.316	−0.480	−0.153	<0.0005
Amount of daily maize silage (kg)	1.008	0.008	0.004	0.013	<0.0005
Sudden feed changes					
No	Reference	Reference			
Yes	0.901	−0.104	−0.172	−0.036	0.003
Body condition score	2.151^*β*^	0.766^*β*^	0.426^*β*^	1.106^*β*^	<0.0005^*β*^
condition score squared	0.878^*β*^	−0.130^*β*^	−0.203^*β*^	−0.057^*β*^	0.001^*β*^
Days in milk	0.998^*β*^	−0.002^*β*^	−0.002^*β*^	−0.001^*β*^	<0.0005^*β*^
Days in milk squared	1.000^*β*^	1.50^−06*β*^	1.06^−06*β*^	1.93^−06*β*^	<0.0005^*β*^
Normal appetite					
No	Reference	Reference			
Yes	2.018	0.702	0.433	0.971	<0.0005
Pregnant					
No	Reference	Reference			
Yes	0.766	−0.267	−0.323	−0.211	<0.0005
Subclinical mastitis					
Negative	Reference	Reference			
Positive	0.940	−0.062	−0.126	0.001	0.055
Constant	1.289	0.254	−0.199	0.706	0.272

^*β*^Variable is part of a curvilinear relationship, and therefore coefficients cannot be interpreted in isolation but rather in combination with the other relevant coefficients for the curvilinear variable, and these combinations are best reported using a graph (Figures 3–5).

**Table 6:** Final generalized linear mixed regression model for natural log of daily milk production for 1458 cow-visit observations from 607 farm visits of 235 cows on 80 smallholder dairy farms near Meru, Kenya, in 2016–2017, adjusting for clustering of cows within farms.

Variables and their categories	Exponentiated coefficient	Coefficient	(95% conf. interval)		*P* value
Amount of daily *Calliandra/Sesbania* (kg)	1.094	0.090	0.012	0.168	0.024
Visit number^*α*^	1.009 ^*α*^	0.009^*α*^	0.003^*α*^	0.015^*α*^	0.002^*α*^
Amount of daily dairy meal (kg)	1.047	0.046	0.027	0.065	<0.0005
Amount of daily maize germ (kg)	0.811	−0.210	−0.363	−0.058	0.007
Amount of daily maize silage (kg)	1.008	0.008	0.004	0.012	<0.0005
Napier grass fed					
No Napier grass fed	Reference	Reference			
Fed at any height	1.076	0.073	0.016	0.130	0.012
Sudden feed changes					
No	Reference	Reference			
Yes	0.901	−0.104	−0.162	−0.046	<0.0005
Body condition score	2.038^*β*^	0.712^*β*^	0.378^*β*^	1.045^*β*^	<0.0005^*β*^
Body condition score squared	0.886^*β*^	−0.121^*β*^	−0.193^*β*^	−0.050^*β*^	0.001^*β*^
Days in milk	0.998^*β*^	−0.002^*β*^	−0.002^*β*^	−0.001^*β*^	<0.0005^*β*^
Days in milk squared	1.000^*β*^	1.59^−06*β*^	1.15^−06*β*^	2.02^−06*β*^	<0.0005^*β*^
Normal appetite					
No	Reference	Reference			
Yes	1.377	0.320	0.097	0.542	0.005
Pregnant					
No	Reference	Reference			
Yes	0.742	−0.299	−0.353	−0.245	<0.0005
Constant	1.730	0.548	0.120	0.975	0.012

^*α*^Ordinal variable: time of farm visit modeled as a continuous variable. ^*β*^Variable is part of a curvilinear relationship, and therefore coefficients cannot be interpreted in isolation but rather in combination with the other relevant coefficients for the curvilinear variable, and these combinations are best reported using a graph (Figures 4 and 5).

## Data Availability

The field data used to support the findings of this study are available from the corresponding author upon reasonable request.

## References

[1] Bindari N. N., Yugal, Enyenihi G. E., Akpabio U., a Offiong E. E. (2013). Effects of nutrition on reproduction : a review. *Advances in Applied Science Research*.

[2] Muraya J. (2018). Cross-sectional study of productive and reproductive traits of dairy cattle in smallholder farms in Meru, Kenya. *Livestock Research for Rural Development*.

[3] Blake R. W. Dairy cattle response in difficult environments.

[4] Njarui D. M. G., Gatheru M., Wambua J. M., Nguluu S. N., Mwangi D. M., Keya G. A. (2011). Feeding management for dairy cattle in smallholder farming systems of semi-arid tropical Kenya. *Livestock Research for Rural Development*.

[5] Bii B. (2017). *Dairy Farmers Face High Cost of Feeds as Drought Bites - Daily Nation*.

[6] Kirui O. K., Okello J. J., Nyikal R. A. Awareness and use of m-banking services in agriculture: the case of smallholder farmers in Kenya.

[7] Lukuyu B., Kugonza J., Wabwire R., Baltenweck I. Characterisation of the livestock production system and potential for enhancing productivity through improved feeding at Namayumba, Wakiso district of Uganda , March 2011 Farming system.

[8] Muriuki H. G. (2003). *Milk and Dairy Products, Post-harvest Losses and Food Safety in Sub-Saharan Africa and the Near East: A Review of the Small-Scale Dairy Sector-Kenya*.

[9] Afande F. O., Wachira I. J. (2015). Constraints to profitability of smallholder dairy farmers in nyeri south sub-county, Kenya. *Developing Country Studies*.

[10] University of Missouri Center (2013). Chapter 1 : defining agroforestry. *Training Manual for Applied Agroforestry Practices*.

[11] Cuddeford V. (1999). *A Fodder Hedge Provides Feed for Cattle in the Dry Season - Barza Scripts*.

[12] Sibanda H. M., Ndlovu N. R. The value of indigenous browseable tree species in livestock production in semi-arid communal grazing areas of Zimbabwe.

[13] Franzel S., Carsan S., Lukuyu B., Sinja J., Wambugu C. (2013). Fodder trees for improving livestock productivity and smallholder livelihoods in Africa,. *Current Opinion in Environmental Sustainability*.

[14] Place F., Roothaert R., Maina L., Franzel S., Sinja J., Wanjiku J. (2009). *The Impact of Fodder Trees on Milk Production and Income Among Smallholder Dairy Farmers in East Africa and the Role of Research Undertaken by the World Agroforestry Centre, Ist.*.

[15] Kamanzi M., Mapiye C. (2012). Feed inventory and smallholder farmers’ perceived causes of feed shortage for dairy cattle in Gisagara District, Rwanda. *Tropical Animal Health and Production*.

[16] Makau D. N., VanLeeuwen J. A., Gitau G. K. (2018). Animal and management factors associated with weight gain in dairy calves and heifers on smallholder dairy farms in Kenya. *Preventive Veterinary Medicine*.

[17] Makau D. N. (2019). Effects of Calliandra and Sesbania supplementation on weight gain in dairy calves on smallholder farms in Kenya. *Preventive Veterinary Medicine*.

[18] Paterson R. T., Kiruiro E., Arimi H. K. (1999). Calliandra calothyrsus as a supplement for milk production in the Kenya highlands. *Tropical Animal Health and Production*.

[19] Devendra C. (1992). *Nutritional Potential of Fodder Trees and Shrubs as Protein Sources in Ruminant Nutrition*.

[20] Trees for the Future (2016). *Why and How Forest Gardens Must Be Used to Improve Livestock Rearing Practices, Reverse Land Degradation, and Increase Smallholder Income*.

[21] Heinrichs J., Jones C. M., Ishler V. A. (2016). Body Condition Scoring as a Tool for Dairy Herd Management. https://extension.psu.edu/body-condition-scoring-as-a-tool-for-dairy-herd-management.

[22] Richards S. M. (2017). *Productivity and Welfare of Cows on Smallholder Dairy Farms in Kenya*.

[23] Cook B. (2005). *Calliandra calothyrsus*. *Tropical Forages: An Interactive Selection Tool*.

[24] Kathambi E. K., Van Leeuwen J. A., Gitau G. K., Mckenna S. L. (2018). A cross-sectional study of the welfare of calves raised in smallholder dairy farms in Meru, Kenya, 2017. *Veterinary World*.

[25] Lukuyu B., Gachuiri C. K., Lukuyu M. N., Lusweti C., Mwendia S. (2012). *Feeding Dairy Cattle in East Africa*.

[26] Van Der Heijden G. J. M. G., Donders A. R. T., Stijnen T., Moons K. G. M. (2006). Imputation of missing values is superior to complete case analysis and the missing-indicator method in multivariable diagnostic research: a clinical example. *Journal of Clinical Epidemiology*.

[27] Kincaid C. Guidelines for selecting the covariance structure in mixed model analysis.

[28] Dohoo I. R., Martin S. W., Stryhn H. (2009). *Repeated Measures Data*.

[29] Richards S. (2016). Randomized controlled trial on impacts of dairy meal feeding interventions on early lactation milk production in smallholder dairy farms of Central Kenya. *Preventive Veterinary Medicine*.

[30] Changwony K., Kitilit J. K. (2014). *Dairy Cattle Feed Types , Quantity Fed and Their Effects on Milk Density within Bomet, Bureti and Nyamira Districts*.

[31] Delaby L., Husson F., Faverdin P. (2010). Predicting energy × protein interaction on milk yield and milk composition in dairy cows. *Journal of Dairy Science*.

[32] Romney D. Technology development and field testing: access to credit to allow smallholder dairy farmers in Central Kenya to reallocate concentrates during lactation.

[33] Oetzel G. R. Subacute ruminal acidosis in dairy Herds : physiology , pathophysiology , milk fat responses , and nutritional management.

[34] Yitbarek M. B., Tamir B. (2014). Silage additives: review. *Open Journal of Applied Sciences*.

[35] Kordi M., Naserian A. A. (2012). Influence of wheat bran as a silage additive on chemical composition , in situ degradability and in vitro gas production of citrus pulp silage. *African Journal of Biotechnology*.

[36] Qin M.-z., Shen Y.-x. (2013). Effect of application of a bacteria inoculant and wheat bran on fermentation quality of peanut vine ensiled alone or with corn stover. *Journal of Integrative Agriculture*.

[37] Smith T. (2010). Some tools to combat dry season nutritional stress in ruminants under african conditions. *Development and Field Evaluation of Animal Feed Supplementation Packages*.

[38] Rengman S., Johansson B., Murphy M. Dietary nutrient density and effects on intake and production.

[39] Moran J. (2005). Supplements for milking cows. *Tropical Dairy Farming : Feeding Management for Small Holder Dairy Farmers in the Humid Tropics*.

[40] Karuga J. (2011). *Napier Grass Farming Method Promises Higher Milk Yield*.

[41] Moran J. (2005). Problems with unbalanced diets. *Tropical Dairy Farming: Feeding Management for Small Dairy Farmers in the Humid Tropics*.

[42] De Vries M. J., Veerkamp R. F. (2000). Energy balance of dairy cattle in relation to milk production variables and fertility. *Journal of Dairy Science*.

[43] Silvestre A. M., Martins A. M., Santos V. A., Ginja M. M., Colaço J. A. (2009). Lactation curves for milk, fat and protein in dairy cows: a full approach. *Livestock Science*.

[44] Macciotta N. P. P., Dimauro C., Rassu S. P. G., Steri R., Giuseppe P. (2011). The mathematical description of lactation curves in dairy cattle. *Italian Journal of Animal Science*.

[45] Johnson C. R., Lalman D. L., Brown M. A., Appeddu L. A., Buchanan D. S., Wettemann R. P. (2003). Influence of milk production potential on forage dry matter intake by multiparous and primiparous Brangus females1, 2. *Journal of Animal Science*.

[46] Smith J. F., Brouk M. J. (2000). Factors affecting dry matter intake by lactating dairy cows. *Kansas Agricultural Experiment Station Research Reports*.

[47] Olori V. E., Brotherstone S., Hill W. G., McGuirk B. J. (1997). Effect of gestation stage on milk yield and composition in Holstein Friesian dairy cattle. *Livestock Production Science*.

[48] Bohmanova J., Miglior F., Kelly M., Kistemaker G., Loker S. (2006). *Effect of Pregnancy on Milk Yield of Canadian Dairy Cattle*.

[49] Penasa M., De Marchi M., Cassandro M. (2016). Short communication: effects of pregnancy on milk yield, composition traits, and coagulation properties of Holstein cows. *Journal of Dairy Science*.

[50] Gonçalves J. L. (2018). Bovine subclinical mastitis reduces milk yield and economic return. *Livestock Science*.

[51] Muraya J. (2019). *Improving Productivity and Reproductive Efficiency of Smallholder Dairy Cows in Kenya*.

[52] Bhide A., Shah P. S., Acharya G. (Apr. 2018). A simplified guide to randomized controlled trials. *Acta Obstetricia et Gynecologica Scandinavica*.

[53] Desaeger J., Rao M. R. (1999). The root-knot nematode problem in sesbania fallows and scope for managing it in western Kenya. *Agroforestry Systems*.

